# Macrophage Origin, Metabolic Reprogramming and IL-1β Signaling: Promises and Pitfalls in Lung Cancer

**DOI:** 10.3390/cancers11030298

**Published:** 2019-03-02

**Authors:** Emma Guilbaud, Emmanuel L. Gautier, Laurent Yvan-Charvet

**Affiliations:** 1 Institut National de la Santé et de la Recherche Médicale (Inserm) U1065, Université Côte d’Azur, Centre Méditerranéen de Médecine Moléculaire (C3M), Atip-Avenir, Fédération Hospitalo-Universitaire (FHU) Oncoage, 06204 Nice, France; eguilbaud@unice.fr; 2 Institut National de la Santé et de la Recherche Médicale (Inserm) UMR_S 1166, Sorbonnes Universités, Hôpital de la Pitié Salpêtrière, 75013 Paris, France; emmanuel-laurent.gautier@inserm.fr

**Keywords:** lung adenocarcinoma, macrophage, immunotherapy, interleukin-1β and immunometabolism

## Abstract

Macrophages are tissue-resident cells that act as immune sentinels to maintain tissue integrity, preserve self-tolerance and protect against invading pathogens. Lung macrophages within the distal airways face around 8000–9000 L of air every day and for that reason are continuously exposed to a variety of inhaled particles, allergens or airborne microbes. Chronic exposure to irritant particles can prime macrophages to mediate a smoldering inflammatory response creating a mutagenic environment and favoring cancer initiation. Tumor-associated macrophages (TAMs) represent the majority of the tumor stroma and maintain intricate interactions with malignant cells within the tumor microenvironment (TME) largely influencing the outcome of cancer growth and metastasis. A number of macrophage-centered approaches have been investigated as potential cancer therapy and include strategies to limit their infiltration or exploit their antitumor effector functions. Recently, strategies aimed at targeting IL-1β signaling pathway using a blocking antibody have unexpectedly shown great promise on incident lung cancer. Here, we review the current understanding of the bridge between TAM metabolism, IL-1β signaling, and effector functions in lung adenocarcinoma and address the challenges to successfully incorporating these pathways into current anticancer regimens.

## 1. Introduction

Lung cancer is the leading cause of cancer-related death and the second most common malignancy with non-small cell lung cancer (NSCLC) referring for lung adeno and squamous carcinomas and accounting for up to 80% of all newly diagnosed lung cancer cases [1,2]. The overall five-year survival rate among newly diagnosed lung cancer patients remains in the low range of 15% [3]. This is in part because (1) the majority of lung cancer cases are diagnosed relatively late in the course of the disease despite advances in lung cancer screening and diagnosis and (2) lung adenocarcinomas are extremely diverse in terms of histopathology, radiology, and molecular spectrum impeding treatments despite multimodality therapeutics [4]. Since the 80’s, macrophage density from biopsies of dozen types of cancer, including lung adenocarcinoma, has been linked to tumor growth and poor outcomes for cancer patients [5,6,7,8]. However, distinct subsets of tumor-associated macrophages (TAMs) exist within tumors and these cells can adopt a wide array of phenotypes depending on their environment. We are just starting to better appreciate the ontogeny and effector functions of these TAMs and how they can influence the initiation and growth of the tumor depending on a dynamic equilibrium influenced by the tumor microenvironment (TME) [5,6,9,10,11].

The plasticity of macrophages has now been largely accepted [12] and is reflected by their ability to sense, respond, and rapidly adapt to their local environment [13,14,15,16,17] to maintain tissue integrity and preserve self-tolerance [18,19,20]. However, when the host is chronically challenged, upon chronic exposure to irritant particles or infection, for example, macrophages may play a detrimental role contributing to a low-grade inflammatory state that leads to disease progression or even cancer initiation [21,22]. This is illustrated by the increased lung cancer risk in smokers and patients with chronic obstructive pulmonary disease (COPD).

The origin of TAMs within lung adenocarcinoma and their selective functions are currently a topic for debate but a mixed ontogeny and immunosuppressive functions are emerging depending of the stage and location of the tumors [23,24,25,26]. For instance, tumors can early on secrete the colony-stimulating factor 1 (CSF-1 or M-CSF) that expands the pool of macrophages towards the cancer supporting TAM phenotype and later on the chemokine (C-C motif) ligand 2 (CCL2) that attracts monocytes [5,6,9,10,11]. Once infiltrated within the tumor, TAMs maintain intricate interactions with malignant cells within the TME and this is most likely the key culprit of their antitumoral response largely influencing the outcome of tumor growth and metastasis.

In this review, we describe recent advances made on the ontogeny of lung resident macrophages and their expansion and metabolic rewiring towards the lung cancer supporting TAM phenotype, which depend on a specialized TME. We discuss therapeutic promises of general therapies to block macrophage recruitment to tumors or more selective therapies to reeducate their tumoricidal functions, both having reached clinical trials. We further outline the contribution of the IL-1β signaling pathway, and how its metabolic-dependent modulation in TAMs could explain part of the anti-tumorigenic potential of IL-1β inhibition.

## 2. Environment-Dependent Maintenance of Lung Macrophages during Homeostasis

### 2.1. Lung Macrophage Ontogeny and Maintenance

**Figure 1:**
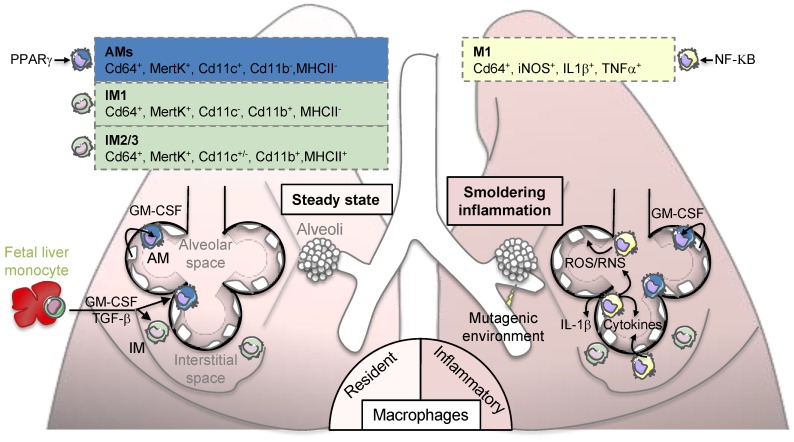
Lung macrophage origin and contribution to “smoldering inflammation”. Left panel: lung-resident macrophages are derived from fetal liver monocytes originating during embryogenesis. The genesis and self-maintenance of macrophages depend on granulocyte-macrophage colony-stimulating factor (GM-CSF) and transforming growth factor β (TGF-β). Four populations of macrophages are present in the lung and are defined by their locations and expression of specific cell surface markers (please refer to boxes): alveolar macrophages (AMs) reside in the airspaces of lung where they self-renew thanks to GM-CSF-expressing alveolar cells. AMs express peroxisome proliferator-activated receptor γ (PPARγ) to maintain lipid homeostasis most likely required for surfactant lipid recycling. Three interstitial macrophage (IMs) populations are located in the lung interstitium and have potential immunoregulatory properties. Right panel: upon exposure to irritant particles or chronic inflammation, macrophages can be primed into an inflammatory M1 phenotype participating to a “smorldering inflammation”. This inflammation is illustrated by the secretion inflammatory cytokines such as interleukin-1β (IL-1β) or tumor necrosis factor α (TNFα) that are under the control of the transcriptional factor NF-kB and the production of reactive oxygen or nitrogen species (ROS/RNS) that favor the induction of somatic mutations in surrounding epithelial cells.

Macrophages are tissue-resident cells that act as immune sentinels to maintain tissue integrity, preserve self-tolerance, and protect against invading pathogens [18,19,20]. In the lung, early preclinical studies have suggested that monocytes poorly contribute to tissue-resident macrophages at steady state and their maintenance mainly relies on homeostatic self-renewal [27,28,29]. Modern tools using fate mapping and tracing methods in mice have confirmed that a specific population of lung alveolar macrophages (AMs) originates from fetal liver progenitors and relies in large part on their ability to self-renew at steady state [30,31,32,33,34,35,36]. Alveolar macrophages remain the main macrophage population investigated in the lung and reside in the airspace lumen where they are specialized in recycling surfactant molecules and clearing inhaled particles and debris [37,38,39,40,41]. Alveolar macrophages are long-lived, with a turnover rate of only approximately 40% in a year [41]. By contrast to most of tissue-resident macrophages, the maintenance of AMs is not supported by CSF-1 as illustrated in op/op mice that harbor a mutation in this gene [18,19,20]. Indeed, mouse AMs are highly dependent on the granulocyte-macrophage colony-stimulating factor (GM-CSF) and the transforming growth factor beta (TGF-β) for their genesis and survival [42,43]. Three additional subpopulations of mouse interstitial macrophages (IMs) have been identified in the pulmonary interstitium, comprising up to 4% of lung macrophages and presumably existing between the blood compartment and the airways [44,45]. These macrophage populations are defined by their location and site of origin, and distinguished by specific cell surface markers (Figure 1, left panel) [46]. As for AMs, two populations of mouse IMs may self-maintain independently of adult hematopoiesis [44,45]. Emphasizing the complexity of IMs, the third population of IMs could be maintained by circulating monocytes to exert their tissue remodeling and immunoregulatory activities [47,48,49]. Consistently, there is a strong interest to develop new tools to specifically target these different macrophage populations in vivo and address their transcriptional signature and immune function during lung homeostasis and diseases [50].

### 2.2. Environment-Dependent Lung Macrophage Identity during Homeostasis

The plasticity of macrophages has now been largely accepted [12] and is reflected by their ability to sense, respond and rapidly adapt to their local environment, including inflammatory signals [13], ectopic nutrient deposition [14,15] or apoptotic debris [16,17]. Consistently, gene expression patterns of mouse macrophages are diverse among various peripheral tissues reflecting their propensity to sense environmental cues and the wide array of phenotypes they can adopt [51,52,53]. For instance, macrophages within the distal airways face around 8000–9000 L of air every day and for that reason are continuously exposed to a variety of inhaled particles, allergens or airborne microbes [37,38,39,40]. A handy and consequently persistent shorthand for understanding macrophage function divides these cells into two extreme phenotypes of a large spectrum in vitro by polarizing them into a pro-inflammatory M1 phenotype induced by LPS (alone or in combination with INFγ) or an alternative M2 phenotype induced after IL-4 stimulation [54,55,56]. Although oversimplified, this in vitro classification has provided a useful guide for investigating the mechanisms that dictate macrophage switch during lung inflammation or repair [57,58]. For instance, while the inflammatory properties of M1 macrophages contribute to early host defense or injury responses, the repair functions of M2 macrophages play a crucial role during wound healing. This adaptation to the environment could explain why macrophage effector functions is intimately linked to intracellular metabolic reprograming to rapidly respond to the adequate energy demand [59,60,61,62]. However, this classification limits our ability to clearly define a boundary and categorize cells during homeostasis as macrophages respond to a vast number of steady state single or combined environmental cues, thus complicating our understanding of the precise mechanisms and metabolic flows that maintain macrophage basic cellular functions in vivo. At least, compared to other tissue resident macrophages, AMs are better equipped with genes involved in gas exchange such as the carbonic anhydrase Car4 due to their proximity to the airways or genes regulating lipid metabolism in order to catabolize the surfactant, which are in large part controlled by the transcription factor peroxisome proliferator-activated receptor γ (PPARγ) [51,53]. Consistently, PPARγ expression is important to maintain lung macrophage transcriptome, functionality, and surfactant catabolism as well as response to infection in mice [63,64,65,66]. This tissue specific transcriptional signature is in part the consequence of a chromatin landscape reshaping induced by the local microenvironment [53] that could largely be influenced by acute and chronic inflammation [57,67] and epigenetic-dependent reprogramming [67,68]. In contrast, little is known about the regulation of IM populations. Besides sensing microbial products [47], IMs may be impacted by low oxygen tension (i.e., hypoxia) to exert their immunoregulatory activities in mouse pulmonary hypertension or allergenic contexts [69,70]. Thus, given their different phenotypes and ontogenies, it is now clear that AMs and IMs perform different functions in the lung that are so far been linked to tissue maintenance (i.e., surfactant catabolism and luminal infection) and innate immunoregulatory mechanisms (i.e., hypoxia and allergen sensing and tissular infection).

## 3. Lung Macrophage Origin and Diversity in Lung Adenocarcinoma

### 3.1. Smoldering Inflammation

Environmental and genetic factors influence lung cancer pathogenesis with cigarette smoke being the major environmental risk factor, followed by chronic infection and dietary factors. Chronic inflammatory diseases are linked to the initiation of tumorigenesis in part by creating a mutagenic environment in sub-epithelial stroma [21,22]. This type of inflammation with increased cancer risk is often referred to “smoldering inflammation”. Immune cells, especially macrophages, participate to the mutagenic environment by producing various cytokines (including IL-1β) and generating reactive nitrogen and oxygen species that promote genetic instability and induce somatic mutations in epithelial cells (Figure 1, right panel). Even if detailed mechanisms underlying tobacco-induced cancerogenesis are not completely elucidated [71], there is strong evidence that cigarette smoke contributes to this smoldering inflammation by inducing secretion of inflammatory cytokines and macrophage apoptosis [72,73] along with the formation of lung nodules [74]. The two most frequent oncogenic mutations in lung adenocarcinoma, which are generally mutually exclusive, include the activating mutations in a small GTPase transductor protein KRAS (V-KI-ras2 Kirsten rat sarcoma viral oncogene homolog) and the epidermal growth factor receptor (EGFR) [4]. In preclinical and clinical studies, chronic obstructive pulmonary disease (COPD) leads to increased lung cancer risk [75,76]. This disease is predicted to rank in the top five of overall burden of disease by 2020 according to the World Heathy Organization (WHO) [77] because of increased tobacco use and its relationship to the metabolic syndrome [78,79]. There are also evidences that COPD could be driven by chronic exposure to irritant particles such as asbestos or silica through NOD-like receptor family, pyrin domain containing 3 (NLRP3) inflammasome-dependent IL-1β secretion [80,81]. Thus, chronic inflammatory diseases, dominated by macrophage inflammation, is a culprit of cancer initiation.

### 3.2. Resident Tumor-Associated Macrophages (rTAMs)

**Figure 2:**
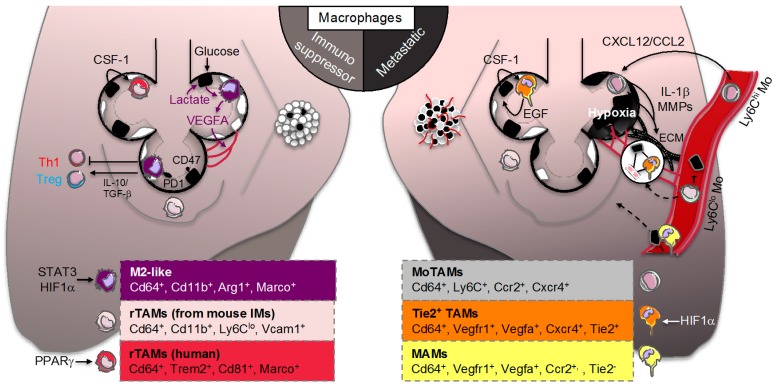
Macrophage effector functions as part of the 7th hallmark of cancer. Right panel: In established tumors, tumor-associated macrophages (TAMs) are the major part of the immune infiltrate that constitutes the tumor microenvironment (TME). Malignant cells produce the colony stimulating factor 1 (CSF-1), which participates to the conversion of tissue-resident macrophages into resident-TAMs (rTAMs). Their origin and cell surface makers may differ between mice and humans, with PPARγ being highly expressed in human rTAMs. Tumor cells also produce lactate through anaerobic glycolysis referred as the “Warburg effect” that can feed cancer cells in a cell-autonomous fashion for proliferation or act in a paracrine fashion to stabilize the hypoxia-inducible factor 1α (HIF1α) and promote a non-classical “M2-like” macrophage polarization. The signal transducer and activator of transcription 3 (STAT3) is another key transcription factor of M2 polarization. These M2-like macrophages participate to the tumor growth through at least 4 mechanisms: (1) secretion of the angiogenic vascular endothelial growth factor A (VEGFA), (2) expression of the immune checkpoint programmed death-1 (PD-1), (3) defect in recognizing and phagocytosing CD47-expressing tumor cells and (4) immunosuppression through inhibition of Th1 helper cells (Th1) and recruitment of regulatory T cells (Treg). Left panel: TAMs are also involved in more chaotic metastatic tumors. A feed-forward loop between CSF-1-expressing tumor cells and EGF-expressing TAMs contributes to intensive proliferation and oxygen consumption leading to a hypoxic environment. Tumor cells also secrete chemokine ligands such as CXCL12 and CCL2, involved in the recruitment into the tumor site of newly monocyte-derived TAMs (MoTAMs) from circulating Ly6C^hi^ monocytes contributing to the expansion of the tumor and the hypoxic niche. Hypoxia within tumor nest alters tumor cells and surrounding MoTAMs promoting extracellular matrix (ECM) remodeling through secretion of IL-1β and metalloproteases (MMPs). This remodeling favors the “angiogenic switch”. A population of Tie2^+^ TAMs, which most likely derives from a subpopulation of circulating Ly6C^lo^ monocytes, is located within the tumor vasculature interacting with mammalian-enabled (MENA)-expressing tumor cells and endothelial cells to further promote angiogenesis and create a metastatic environment. Circulating Ly6C^lo^ monocytes also scavenge tumor materials to prevent tumor invasion whereas metastasis-associated macrophages (MAMs) allow the extravasation of tumor cells into the lung.

On the site where a tumor develops, malignant cells are surrounded by non-malignant stroma cells that are part of the TME. Non-malignant populations include connective tissue cells and leucocytes, with TAMs representing the majority of the leukocyte population [82,83]. The specific maintenance of tissue-resident macrophages through in situ proliferation and the diversity of TAMs within tumors have challenged our understanding of their role in tumors. Indeed, in mouse models of brain tumors and pancreatic cancer, TAMs can originate from both circulating monocytes and tissue-resident macrophages where they could facilitate tumor growth by contributing to tissue remodeling [25,84]. After inoculation of TC-1 lung carcinoma cells in mice, the group of Boissonnas identified a specific population of mouse rTAMs that originated from tissue-resident IMs already present in healthy lungs that could support lung tumor development (Figure 2, left panel) [85]. In comparison to other macrophage subsets, profiling of rTAMs revealed a transcriptional signature associated to tissue remodeling including transcripts related to extracellular matrix (ECM) and vasculature interactions that supported tumor cell growth [85]. Using single-cell and mass cytometry by time of-flight (CyTOF) analyses in early human lung adenocarcinomas, the group of Merad also identified a unique tumor-specific macrophage population that dissociated from tissue-resident macrophages (Figure 2, left panel) [86]. These rTAMs exhibited an upregulation of transcripts involved in macrophage effector functions such as triggering receptor expressed on myeloid cells-2 (TREM2), tetraspanin CD81 or macrophage receptor with collagenous structure (MARCO) that were associated with a significant survival disadvantage [86]. As reviewed elsewhere, it was previously reported that both TREM2 and MARCO are critical in mouse airway macrophages to limit pro-inflammatory or Toll-like receptor responses [41]. Intriguingly, these human rTAMs also expressed higher levels of the transcription factor PPARγ involved in tissue-resident AM immunomodulatory functions and surfactant catabolism [86]. These findings suggest that rTAMs signature may be associated with a tumor-specific metabolic rewiring opening therapeutic perspectives for lung cancer diagnosis and treatment [87]. For instance, pro-surfactant protein B (SFTPB), known to be transcriptionally controlled by PPARγ, is used as a serum biomarker of lung adenocarcinoma both in preclinical and clinical studies [88,89,90]. Additionally, higher density of anti-tumoral TAMs were observed in human lung tumor nests and stroma [21] and stroma TAMs were associated with systemic blood inflammation (i.e., elevated plasma CRP levels), adverse prognostic factors (i.e., lymph node metastasis) or poor overall survival [91,92].

### 3.3. Monocyte-Derived TAMs (MoTAMs)

**Figure 3:**
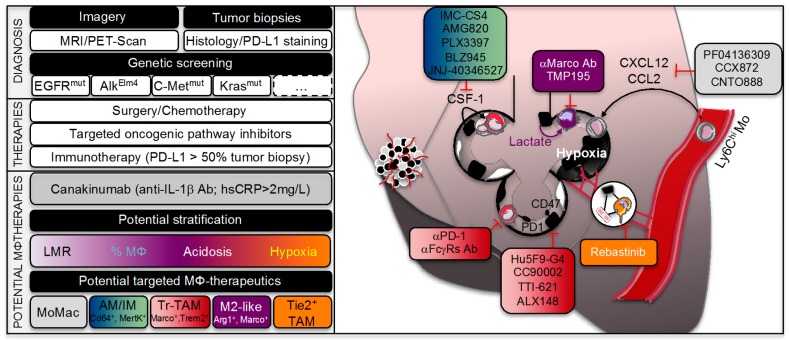
Lung adenocarcinoma treatment and emerging therapeutic potential of targeting macrophages. Diagnosis of lung adenocarcinoma patient requires at first magnetic resonance imaging (MRI) or positron-emission tomography scan (PET-scan). Tumor biopsies were also performed to further characterize the histology of the tumor and to determine the cancer cell’s origin, the disease progression, and the expression of PD-L1 among other features. Oncogenic mutations driving lung adenocarcinomas were screened, of which V-KI-ras2 Kirsten rat sarcoma viral oncogene homolog (KRAS) and epidermal growth factor receptor (EGFR) mutations were the most frequent. These different diagnoses allow personalized treatment options with targeted oncogenic pathway inhibitors and/or chemotherapy. Immunotherapy is highly patient-dependent since the treatment with a checkpoint inhibitor that targets the PD-1/PD-L1 pathway requires tumors expressing levels of PD-L1 higher than 50%. These therapies are generally not exclusive and different strategies are employed for a better healing without remission. Novel potential therapies are aimed at targeting tumor-associated macrophages (TAMs). Whether or not IL-1β inhibitory antibody (Canakinumab) targets macrophages, its use on patients with C-reactive protein (CRP) levels higher than 2 mg/L reduced the rate of lung cancer. Composition of the tumor microenvironment (TME) may allow patient stratification. For instance, lymphocyte-monocyte ratio (LMR), a prognostic factor and a predictor survival, could be modified with CCR2 inhibitors (PF04136309 or CCX872) or with CCL2 inhibitors (CNTO888), preventing the recruitment of circulating Ly6C^hi^ monocytes into tumors. To limit the conversion of tissue-resident macrophages (i.e., alveolar macrophages AMs and interstitial macrophages IMs) into TAMs, blocking antibodies anti-CSF-1R (IMC-CS4 or AMG820) and tyrosine kinase inhibitors (PLX3397, BLZ945 or JNJ-40346527) are used. Cancer cells express CD47 on their surface, known to be a “don’t eat me” signal and recognized by SIRP1α expressed on macrophages, which triggers a cascade of events that inhibit phagocytosis: anti-CD47 (Hu5F9-G4 or CC90002) or competitive recombinant SIRP1αFC (TTI-621 or ALX148) are developed as a way to reeducate TAMs for eliminating cancer cells. As for T-cells, TAMs also express the immune checkpoint receptor PD-1, inducing immune tolerance and TAMs PD-1 expression reduced the phagocytic potency against tumor cells. Immunotherapy with the use of αPD-1 not only targets PD-1/PD-L1 pathway on T-cells but is efficient to reactivate phagocytic potency of macrophages. However, TAMs could limit anti-PD-1 therapeutic benefits by stealing and capturing αPD-1 antibody from the CD8+ T-cells via FcγRIIb/III receptors unless if αFcγRs antibodies are administrated before. Another way to reeducate TAMs is to convert M2-like macrophages to an antitumor phenotype in targeting MARCO (αMarco Ab) or in inhibiting histone deacetylase (TMP195) to reprogram macrophage-dependent T-cell immune responses. Rebastinib reduced cancer cell metastasis by inhibiting a specific Tie2^+^ TAMs population implicated in the angiogenic switch.

While the role of rTAMs in promoting cancers spread (i.e., metastasis) is well documented, the role of MoTAMs remains much less understood beyond their potential roles in continuously replenishing tumors [6,93]. The TAMs were originally hypothesized to originate from circulating monocytes that were recruited in response to chemotactic signals released from tumor cells with a subset of these cells being called myeloid-derived-suppressor cells [94,95]. Targeting chemokine interactions and subsequent recruitment of macrophages within tumors, including the CCL2/CCR2 or CXCL12/CXCR4/7 chemokine-chemokine receptor axes, have shown great potential for cancer therapies in various mouse models of cancer metastasis [96,97,98]. In the clinic, antibodies that selectively target CCL2 (CNTO888) have produced mixed results as antitumor activity [99,100,101]. By contrast, CCR2 inhibitors (i.e., PF04136309 or CCX872) are currently tested in metastatic pancreatic cancer (Figure 3) [102,103]. Using, a mouse model of lung cancer metastasis driven by p53 deficiency and the oncogenic mutation *Kras^G12D^* the group of Pittet showed that circulating classical inflammatory monocytes employ the chemokine receptor CCR2 to promote a potent macrophage amplification program that generated TAMs within the lung (Figure 2, right panel) [23,94]. A role for the CXCL12/CXCR4/7 chemokine–chemokine receptor axes has also been proposed in mouse lung cancer metastasis-induced by lung carcinoma cell transplantation [104,105] most likely by shaping infiltrated immune cell population and promoting angiogenesis [106]. By contrast to classical monocytes, the group of Hedrick recently identified that nonclassical “patrolling” monocytes, enriched in the microvasculature of multiple mouse metastatic tumor models, prevented tumor invasion and reduced lung metastasis by scavenging tumor material from the lung vasculature (Figure 2, right panel) [107]. Further investigations will be required to pinpoint at with stage the imbalance between classical and non-classical MoTAMs occurs in lung cancer. Still, some evidences in humans indicate that the lymphocyte–monocyte ratio (LMR) could not only be an independent prognostic factor in patient with NSCLC (Figure 3) [108] but also a predictor of survival and clinical outcome before complete resection for primary lung cancer or after treatment with anti-angiogenic therapy plus chemotherapy [109,110,111,112]. However, whether this ratio predicts a chemokine gradient, a switch between MoTAMs and/or the pathologic role for infiltrated MoTAMs remain to be fully elucidated.

## 4. Lung Macrophage Immunometabolism and Function in Lung Adenocarcinoma

### 4.1. TAM Immunomodulation

Understanding the distinct functions of different TAM populations within tumors remains an intense area of research. Nevertheless, in the vast majority of cancers, macrophages exhibit an overall immunosuppressive phenotype characterized by low levels of inflammatory molecules and an increased expression of transcripts expressed by alternatively activated M2 macrophages (Figure 2, left panel) [6]. Their in vitro and in vivo responses are associated with TGF-β and other growth factor such as vascular endothelial growth factor (VEGFA), interleukin or metalloprotease production that could impact their proliferation and differentiation in an autocrine manner and their surrounding environment in a paracrine manner [113,114]. This macrophage switch was recently highlighted in a transcriptional single cell analysis of human lung adenocarcinomas [115]. This antitumor response is thought to be mediated by the local environment created by the tumor to educate and take advantage of them. Although the M1/M2 distinction is oversimplified, therapies aimed at reprogramming TAMs towards a pro-inflammatory phenotype have emerged as a way to promote tumoricidal functions of TAMs. In animal models, drugs that inhibit key signaling molecules involved in M2 polarization (i.e., IL-4, STAT3 or PI3-kinase) successfully limited the immunosuppressive functions of TAMs and shrank tumors [116,117,118,119,120]. Macrophage-specific deletion of c-MYC also reduced tumor growth by preventing alternative TAM polarization [121]. Similarly, targeting the macrophage receptor with collagenous structure (MARCO), which is a key M2 marker, reprogrammed macrophage-dependent T-cell immune responses restricting tumor development and metastasis in mice (Figure 3) [122]. These findings add on the original Weissman’s work on how cancer cells escape TAM’s cancer-killing potential. Indeed, almost every type of cancer cell expresses CD47 at their cell surface, which is a molecule known for its role on normal, healthy cells as a “don’t eat me” signal to phagocytosing macrophages (Figure 2, left panel) [123]. By expressing CD47, cancer cells will block “eat me” signals (such as the molecule calreticulin, which marks the cells for phagocytosis) by engaging the signal regulatory protein alpha (SIRP1α) on the surface of macrophages and limiting their cellular rearrangement for efficient engulfment. These findings led to novel therapeutic approaches targeting CD47 or SIRP1α (i.e., anti-CD47 antibodies such as Hu5F9-G4 or CC90002 and competitive recombinant SIRP1αFC such as TTI-621 and ALX148) as a way to reeducate TAMs for eliminating cancer cells in humans (Figure 3) [124,125]. Another example to reeducate the tumoricidal activity of TAMs is the use of CD40 agonists [126] that have also found their ways to the clinic [11]. Despite major efforts in precision medicine in the era of personalized medicine [127], identifying the type of cancer and patient population who will benefit the most from these emerging “macro-immunotherapies” is still a matter of intense investigations. At least in mouse models of lung cancer metastasis-induced by lung carcinoma cell transplantation or xenograft, therapeutic targeting of intracellular signaling pathways that regulate the switch between macrophage polarization states or the efferocytic function of TAMs was efficient to promote tumor regression and synergized with checkpoint inhibitor therapy [116,117,124,128,129,130,131].

### 4.2. Adaptation of TAM to the “Warburg Effect”

Although the immunosuppression of TAMs is anticipated to be highly complex and context-dependent, recent evidence suggest that metabolic changes in tumor cells could create a metabolic imbalance within the TME that can significantly impact TAM effector functions [132,133]. This environment is generally characterized by hypoxia and acidosis. The latter arises from the propensity for cancer cells to convert glucose to lactate despite the presence of oxygen, a mechanism originally described by Otto Warburg and referred as the “Warburg effect” [134]. Consistently, positron emission tomography (PET) scans using 18F-labeled 2-deoxyglucose as a non-metabolizable glucose analog light up primary and metastatic mouse and human lung cancers (Figure 3). Additionally, an inverse association between the tumor expression of the hypoxia-inducible factor (HIF)-1α, glucose transporter GLUT1 or the lactate dehydrogenase LDHA and the prognostic of patients was observed in lung adenocarcinoma [135,136,137,138]. Recent works from the group of DeBerardinis elegantly showed that human NSCLC heterogeneously oxidize glucose in the tricarboxylic acid (TCA) cycle [139] and even use a larger amount of lactate in a cell-autonomous fashion [140]. Some preclinical evidence in a lung cancer murine xenograft model suggest that targeting GLUT1 inhibits cancer cell growth [141,142]. In a murine model of *Kras^G12D^*-driven lung cancer, pharmaceutical LDHA inhibition also inhibited both tumor progression and regression [143]. Thus, optimization of GLUT1 (BAY and DRB-18) or LDHA inhibitors may offer novel therapeutic strategies for treating lung cancer [144,145,146]. The importance of this metabolic pathway for TAM effector functions has been revealed by Colegio et al. who demonstrated in a murine model of Lewis lung carcinoma that lactic acid produced by tumors stabilizes HIF1α in TAMs, leading to an “M2-like” phenotype that was independent of IL-4R signaling (Figure 2, left panel) [147]. In other mouse model of cancer, the immunosuppression of TAMs was rather attributed to a role of HIF2α in accelerating tumor burden [148]. These discrepancies may reflect the specific metabolic rewiring of tumors depending on the oncogenic driver mutation, the local TME organization or the tissue where they develop and how TAMs form an alliance with cancer cells for metabolic symbiosis or compete for precious nutrients [132,149,150]. Recently, Carmona-Fontaine et al. showed that the modular organization of hypoxia and acidosis within the mouse TME may not only dictate the metabolic rewiring of TAMs but may also be sufficient to recapitulate their spatial diversity in vitro [151,152]. Although oxidative phosphorylation (OXPHOS) is a hallmark of anti-inflammatory M2 macrophages by contrast to glycolytic M1 macrophages in vitro [59,60] and the high glucose requirement of the tumor competes with the surrounding cells present in the mouse TME [153], reports on the glucose utilization and OXPHOS by TAMs in lung adenocarcinoma remain scarce [133].

### 4.3. TAM Immunometabolism beyond Glycolytic Activity

Besides glucose metabolism, modulation of lipid and amino acid metabolism by tumors could also impact the metabolic flexibility and mitochondrial OXPHOS in TAMs [133,149,150]. Although current knowledge on whether modulation of peripheral lipid flux affects cancer pathogenesis is still elusive and controversial [154], an original study from the group of Hoefler revealed that inhibition of peripheral lipolysis was not sufficient to locally affect tumor burden [155]. Moreover, the importance of the pathophysiological tissue context for cancer growth has recently been highlighted by the metabolic phenotyping of a murine model of *Kras^G12D^*-driven lung tumor revealing that glutamine utilization was minimal in contrast to in vitro culture or other types of cancer [156]. Thus, the origin of alteration of lipid and amino acid metabolism in lung adenocarcinoma remains to be fully understood. On one hand, it could be linked to local acidosis that reprogram mitochondrial metabolism and promote histone deacetylation [157,158,159]. In line with these observations, checkpoint blockade therapy to restore immune cell nutrition restriction, nutritional intervention or treatment with a histone deacetylase inhibitor (TMP195) converted immune cells to an antitumor phenotype in mouse models of cancer metastasis (Figure 3) [153,160,161,162] including a shift from an M2 phenotype to a more efferocytic function of TAMs against cancer cells [160]. Alternatively, there is emerging evidence of local communication between cancer cells and TAMs through energy metabolism-derived (i.e., lipid and amino acid) mediators, referred to nowadays as “oncometabolites” or “metabokines” [163,164,165], even though their identification in lung adenocarcinoma has been limited [156,163,164,165]. At least, alteration of lipid metabolism within different populations of macrophages, especially AMs, in an immunocompetent syngeneic murine model injected with Lewis lung carcinoma cell correlated with selective expression of eicosanoids from both tumor cells and TAMs [166,167]. Although a new fatty acid-synthesis inhibitor (ND-646) was shown to blunt lung tumor growth in xenograft and genetically engineered mouse models of NSCLC [168], it is unknown if this compound may restore the functional polarization of TAMs as it has been shown by metformin in other cancer models [169,170]. Apart from glutamine, other amino acids such as arginine derived from the urea cycle may also be involved in the communication between cancer cells and TAMs. Indeed, knockdown of arginase 1 (ARG1) in macrophages prevented lung tumor growth by limiting the hydrolysis of arginine to ornithine [147]. Most of these metabolic pathways converge to increase localized ROS in cancer cells, which activate signaling pathways and transcription factors such as the transcription factor nuclear factor (erythroid-derived 2)–related factor-2 (NRF2) to promote tumorigenesis [160]. For instance, NRF2 regulates serine biosynthesis in NSCLC to generate NADPH and recycle oxidized glutathione, which is critical for the redox balance of cancer cells. Thus, emerging mechanistic insights linking tumor cells to the metabolic reprogramming of TAMs in lung adenocarcinoma hold promise for novel cancer therapy.

### 4.4. Tie2+ TAMs and Metastasis-Associated Macrophages (MAMs)

From original works by the group of Pollard and others [5,6], it has been well appreciated that, by supporting tumor angiogenesis, TAMs not only supply oxygen, nutrients and growth factors for tumor’s development but also lay out a path for metastatic cells to reach new sites in the body, a process known as the “angiogenic switch” [171]. In the mid-1990s, evidence arose from CSF-1 deficient mice that the maturation and survival of macrophages had to do with cancer’s spread (i.e., metastasis), rather than cancer tumor growth [5,6,9,10,11]. This led to the development of several anti-CSF-1R therapies (including CSF-1R blocking antibodies IMC-CS4 or AMG820 or tyrosine kinase inhibitors such as PLX3397, BLZ945 or JNJ-40346527) for patients with advanced solid tumors refractory to standard therapy (Figure 3) [11]. A specific Tie2-positive TAM population has been identified to mediate tumor angiogenesis and support tumor cell intravasation [172,173]. These TAMs could originate from Tie2-positive monocytes that are a subpopulation of nonclassical “patrolling” monocytes playing a role during mouse tumor neovascularization [174,175,176,177]. Mechanistically, these TAMs secrete VEGFA and proteases that degrade basement membranes of the ECM and participate in the formation of a TME of metastasis that comprises a pyramid-type structure on the vessel wall with mammalian-enabled (MENA)-expressing tumor cells that allow interactions with Tie2+ TAMs and blood vessel endothelial cells (Figure 2, right panel) [172,173]. These interactions suggest cell–cell contact for short-range transmission of growth and survival signals as recently illustrated for macrophage-fibroblast circuit [178] and resembles the paracrine EGF–CSF-1 interactions previously observed between TAMs and tumor cells [5,6]. Hypoxia is a major determinant of angiogenesis and HIF1α in TAMs acts as a major regulator of the “angiogenic switch” by inducing a switch from aerobic to anaerobic metabolism and increasing expression of diverse range of factors including VEGFA [174]. Despite mitigated cancer patient outcomes with VEGFR tyrosine kinase inhibitors or VEGFR2 antibodies [4,179], these findings have set up the stage for therapeutic approaches aimed at reducing cancer cell metastasis using a selective Tie2 inhibitor Rebastinib (Figure 3). In mice, the cooperation of the two oncogenes *Kras* and *Myc* has been recently shown to be required for the “angiogenic switch” and the transition to invasive adenocarcinoma [180]. It will remain to be carefully investigated in lung adenocarcinoma whether these Tie2+ TAMs also impact the epithelial–mesenchymal transition (EMT) that has been linked to invasive potential of various cancer cells [113,114,181,182]. Additionally, another population of TAMs seeding at distant sites and being recruited by CCL2 were dubbed metastasis-associated macrophages (MAMs) (Figure 2, right panel). These MAMs allow the extravasation of mouse tumor cells by secreting the chemokine ligand CCL3 and CSF-1 that facilitates metastatic seeding of breast cancer cells in the lung [183,184] and potentially VCAM-1 that transmits survival signals to these tumor cells [185]. The relevance of these MAMs in helping cancer cells to leave blood vessels and promote lung adenocarcinoma metastasis in mice was previously illustrated through their recruitment to extravasating pulmonary metastatic cells regardless of species of origin [186].

### 4.5. Bone Marrow Macrophages and Bone Metastasis

Osteolytic bone metastasis is a frequent event in late stage of lung cancer and is associated with high mortality of lung adenocarcinoma [187,188,189]. Once metastatic tumor cells reach the bone marrow (BM) after adhesion to the mineralized matrix through their invadopodia [190], they promote resorption and bone destruction by interfering with the osteoblastic bone-forming cells and osteoclastic bone-resorbing cells [191]. Osteoclasts are large multinucleated cells that differentiate from macrophage lineage precursors by increasing tartrate-resistant acid phosphatase (TRAP) expression following coregulation by CSF-1 and the receptor activator of nuclear factor κB ligand (RANKL) among other growth factors [192,193]. These growth factors can all be secreted by metastatic lung tumor cells [194,195,196]. Two different populations of macrophage have been described in the BM namely CD11b^hi^ osteomacs [197] and CD11b^int^CD169^+^ macrophages [198] that are localized in different hypoxic area of the BM [199]. Targeting of these macrophage populations influences tumor-induced bone modelling in mouse models of prostate or Lewis lung cancer-induced bone metastasis [200,201]. The underlying mechanisms are not fully understood but could be linked to perturbation of osteoclast differentiation and disruption of the endosteal “osteoblastic niche” through bone resorption (i.e., mineral dissolution, followed by a degradation of the organic phase) and demineralization (i.e., acidification of the extracellular microenvironment and exposure to proteases) [138]. Future studies will be required to investigate whether tumor cells could also locally impact bone tissue, which is a specialized connective tissue consisting of cells and mineralized extracellular matrix (i.e., hydroxyapatite, a type of calcium phosphate) that could be responsible for the production of the calcified matrix in lung nodules [202,203,204].

## 5. IL-1β Signaling and Immunometabolism: A New Role in Lung Adenocarcinoma?

### 5.1. CANTOS Trial and Lung Adenocarcinoma

A recent retrospective analysis by Ridker et al. [205] reveal an unexpected dramatic reduction in the number of incident cases of lung cancer in the large, randomized CANTOS trial (Canakinumab Anti-inflammatory Thrombosis Outcomes Study) originally designed to test the hypothesis that canakinumab, an interleukin 1β (IL-1β) inhibitory antibody, could reduce a secondary cardiovascular event in very high-risk patients with prior myocardial infarction and inflamed setting (i.e., C-reactive protein (CRP) levels > 2 mg/L) (Figure 3). Of note, the incidence rate for all non-lung cancers was not statistically significant and one has to be cautious with hypothesis-based retrospective analysis [206,207]. Nevertheless, these findings led to a follow-up phase I study aimed at testing the combination of canakinumab and PD-1 inhibitor in NSCLC patients. As discussed above the relationship between inflammation and cancer is complicated, probably driven by an immunosuppression within TME. However, the concept of immunotherapy came from Coley’s observation that cancer regression can be achieved by active bacterial infection [208,209]. Thus, the higher incidence of bacterial infection in the CANTOS trial [210] may suggest that by dampening chronic low-grade inflammation, canakinumab has unlocked an unspecific bacterial lung antitumoral activity. However, there are several evidences that blocking IL-1β signaling or upstream NLRP3 inflammasome regulation may have direct anticancer activity [211,212,213,214,215]. First, anti-IL-1β antibody dampens low-grade inflammation [211,216], which could prevent “smoldering inflammation” and reduce the mutagenic environment created by inflammatory immune cells [21,22]. Consistently, expression of specific inflammasome gene modules stratifies older individuals into two extreme clinical and immunological states that was associated with all-cause mortality [217]. Since IL-1β has been linked to airway inflammation [218,219], it will be of interest to know if the high CRP levels in the CANTOS trial at baseline were associated with an incidence of COPD, known to increase lung cancer risk [75,76]. Reduced COPD incidence could partially explain how canakinumab at the 300 mg dose induced a marked separation of the incidence curves for lung cancer within few months. Additionally, deficiency or inhibition of IL-1β signaling in TME has been shown to inhibit tumor angiogenesis and metastasis in mouse models of lung metastases-induced by various metastatic cells regardless of species of origin [220,221,222,223,224,225]. These findings may suggest an additional direct effect of canakinumab on established lung tumors. The CANTOS trial has opened the door to the human relevance of the relationship between IL-1β signaling and lung cancer and provides promising avenue with which to explore new anti-cancer therapies.

### 5.2. IL-1β Signaling and Lung Adenocarcinoma

The cell origin of IL-1β secretion and the implication of NLRP3 or other inflammasomes in lung TME remain to be identified and could have crucial therapeutic implications. Indeed, this pathway could promote on one hand leukocyte priming and trafficking or on the other hand, tumor growth. The tumor growth potential could be attributed to (1) an imbalance between cancer cell proliferation and pyroptosis, (2) an increased epithelial–mesenchymal transition (EMT) through secretion of metalloproteases and other ECM remodeling proteins or (3) an effect on lymphangiogenesis and angiogenesis [211,212,213,214,215]. While macrophages are the main source of IL-1β secretion in the immune response to pathogen [226,227,228], the TME of established tumors is characterized by an immunosuppression dominated by a M2 alternatively activated TAM phenotype that inhibits NLRP3-dependent IL-1β secretion [229]. Consistently, reduced NLRP3-dependent IL-1β secretion is observed in AMs isolated from the bronchoalveolar lavage of lung cancer patients despite a systemic higher NLRP3 inflammasome activation and IL-1β secretion in peripheral blood leukocytes from these patients [230,231]. Thus, the elevated IL-1β concentration in TME [232] could either derive directly from oncogenic lung cancer cells [233] or eventually from another TAM population (i.e., infiltrated MoTAMs) [230]. In the first scenario, combination of canakinumab with anti-PD-1 therapy, as initiated in phase I trial, may be beneficial by dual targeting of cancer and immune cells. However, in the second scenario, outcomes may be mitigated depending on the stage of tumor development and how suppression of an inflammatory response by canakinumab affect the antitumoral anti-PD-1 therapy response [211,212,213,214,215].

### 5.3. Immunometabolism: The Missing Link?

As discussed above, the origin of the tumor immunosuppression could be in part mediated by the hypoxia and acidosis within the TME [147,148], the metabolic restriction imposed by the high energy demand of the tumor [153] or an epigenetic reprogramming of immune cells [160], all these mechanisms being intimately linked to IL-1β secretion [226,234]. However, whether, where and how IL-1β secretion and potentially the activation of the NLRP3 or other inflammasomes could occur in tumor tissues remain elusive. At least, IL-1β secretion in macrophages must be primed by HIF1α and nuclear factor-kappa B (NF-κB)-dependent transcriptional regulation prior cleavage of pro-IL-1β into active IL-1β by caspase 1 [234,235]. Although historically, hypoxia was seen as a main driver of the activation of HIF1α and the expression of glycolytic enzymes to support an anaerobic glycolysis, it has become apparent that other metabolic stimuli can cause HIF1α-dependent metabolic reprogramming, especially in macrophages [59,62]. Are there such metabolic stimuli driving macrophage IL-1β priming in the heterogeneous lung TME [139,156]? As discussed above HIF1α can be stabilized under acidic conditions in TAMs [147]. So, could we envision that IL-1β secretion is dictated by acidosis in TAMs [236]? What we have learned from in vitro studies is that a broken TCA cycle with a concomitant reduction in mitochondrial respiration allows for funneling citrate and succinate out of the mitochondria where succinate acts as an activator of HIF1α turning on the transcriptional expression of pro-IL-1β [237]. Itaconate, one of the most highly induced metabolites in response to pathogens, could be responsible for the upstream regulation of succinate at least in activated macrophages [238]. Thus, it may not be surprising that succinate and itaconate also mediates crosstalk between macrophage metabolism and tumor growth [239,240]. In parallel, perturbed mitochondria metabolism could provide the second signal for proper activation of the inflammasome and subsequent cleavage of pro-IL-1β into active IL-1β by caspase 1 including mitochondrial DNA (mtDNA), calcium and ROS (mROS) among other stimuli [241]. Thus, future studies are warranted to delineate the metabolic communication between cancer cells and macrophages and how it specifically shapes the tumor immune microenvironment response.

## 6. Lung Adenocarcinoma Treatment and Macrophage Interaction

### 6.1. Lung Cancer Therapy Options

In recent years, treatments for all the different hallmarks of cancer have been investigated including anti-proliferative therapies (i.e., tyrosine kinase receptor, cyclin-dependent kinase or growth factor receptor inhibitors), pro-apoptotic therapies (i.e., mitochondrial cell death activation, blockade of DNA repair or telomere destabilization) or anti-angiogenic therapies to limit tumor growth and metastasis [82]. In the context of NSCLC, targeted therapies have been embraced thanks to genetic testing [242]. Targeted therapies prior cytotoxic chemotherapies include EGFR antagonists (i.e., tyrosine kinase inhibitors such as erlotinib, gefitinib, afatinib, necitumumab or osimertinib) for patients harboring an EGFR mutation [243,244,245,246,247,248], ALK inhibitors (i.e., ceretinib, alectinib or crizotinib) for ALK-rearranged NSCLC patients [249] or c-Met inhibitors (i.e., c-Met tyrosine kinase receptor inhibitors) for ALK/c-Met positive NSCLC (Figure 3) [250]. Details on these current therapies are deeply reviewed elsewhere [249,250,251]. However, the majority of lung tumors do not contain identified oncogenic mutations, thereby limiting the use of targeted oncogenic pathway inhibitors to a small fraction of patients (Figure 3). Moreover, no highly effective therapies have been developed for cancers harboring mutant KRAS. For instance, lung cancer patients who are positive for KRAS mutation have a low response rate to EGF tyrosine kinase receptor in part because KRAS and EGFR mutations are generally mutually exclusive and when they co-exist, KRAS mutations may confer resistance to EGFR mutations [4]. Thus, there continues to be a great need for new therapeutic strategies for patients with lung adenocarcinoma. Although several groups have demonstrated that concomitant use of MEK and phosphoinositide 3-kinase (PI3K) inhibitors (MEKi/PI3Ki) can induce dramatic tumor regressions in mouse models of KRAS-mutant non-small cell lung cancer (NSCLC), clinical trials investigating this strategy have been underwhelming [252,253] most likely because of heterogeneity in induction of cancer cell apoptosis [254]. By determining anti-apoptotic addiction, novel BH3-mimetic compounds have been developed to overwhelm anti-apoptotic defense mechanisms in response to oncogenic stress or anti-cancer therapy and a recent study revealed that this treatment could synergize with chemotherapy to induce tumor regression in *Kras^G12D^* mutant lung cancer mouse model [255]. Clinical trials evaluating safety and efficacy of this approach are currently ongoing [252]. Although clinical trials using telomerase inhibitors in NSCLC patients found that overall survival was not improved and this treatment may even causes some adverse decrease in platelet counts [256], they might still be effective in some tumor types (i.e., subgroup of patients with shortened telomers) [257,258] as it was recently observed in preclinical mouse models bearing the *Kras^G12D^* oncogenic mutation [259]. Some drug resistant NSCLC cells could also be sensitized by epigenetic drugs to other cytotoxic drugs [260]. As novel molecular mechanisms of cell death emerge [261], pro-apoptotic therapies may find its path to fight NSCLC, especially KRAS-bearing mutations. Nevertheless, these therapies will have to face drug resistance and find their way within immune checkpoint inhibitors that are increasingly being incorporated into lung cancer treatment protocols.

### 6.2. Emerging Immunotherapy

New paradigms such as targeting tumor immune microenvironments have been tested [262] and from this research, immune checkpoint inhibitors have emerged in the last decade has a new means to treat human cancer. In the context of NSCLC, immunotherapy (i.e., anti-programmed death-1 (PD-1) antibodies such as pembrolizumab or nivolumab and anti-programmed death ligand-1 (PD-L1) antibodies including atezolizumab, durvalumab and avelumab) was used for PD-L1 expression in at least 50% of tumor biopsies (Figure 3) [263,264,265,266]. In preclinical and clinical studies, combined anti-PD-L1 and anti-cytotoxic T-lymphocyte antigen-4 (CTLA-4) monoclonal antibodies may result in higher and more durable responses [267,268]. Even though immune checkpoint inhibitors (anti-CTLA-4 and PD-1/PD-L1 antibodies) have signaled a new direction for lung cancer care, the proportion of patients that respond to these agents remains low and the duration of response is often short [4]. Apart from loss of mismatch-repair function of cancer cells [269], driver mutations could also dampen immune checkpoint blockage by shaping TME. For instance, heterogeneity of intratumor neoantigen or EMT could predict sensitivity to immune checkpoint blockage [270,271] and inactivation of the tumor suppressor liver kinase B1 (LKB1), occurring in one-third of KRAS-mutated lung adenocarcinoma promotes the accumulation of immunosuppressive neutrophils and loss of PD-L1 expression, which is associated with fewer cytotoxic lymphocytes responsible for killing the tumor [272]. Therefore, novel methodologies to enhance the efficacy of immunotherapy in lung cancer are highly desirable.

### 6.3. TAMs and Lung Cancer Therapy Responses

The mechanisms by which TAMs could inhibit antitumor T cell responses involve more than one mechanism. First, TAMs have “tissue-reparative” activity, particularly M2-like macrophages that support tissue remodeling but at the same time suppress type 1 immune response [273]. Indeed, TAMs could inhibit antitumor T cell responses by secreting various factors including interleukin-10, which prevent dendritic cells from activating antitumor T-cell responses [274] or migration inhibitory factor (MIF), TGF-β and amino-acid degrading enzymes such as ARG1 and indoleamine 2,3-dioxygenase 1(IDO1), which promote survival of a subset of anti-inflammatory regulatory T-cells (Figure 2, left panel) [147,274,275,276,277]. The influx of new TAMs to tumors after first chemotherapy could also suppress the cytotoxic activity of antimitotic agents and stimulate tumor relapse [278,279], although they can in some cases be required for optimal therapy [280]. The group of Pittet identified an anti-PD-1 steal mechanism by TAMs which depends on FcγRIIb/III receptors and limits binding and activation to tumor-killing T-cells (Figure 3) [281]. Interestingly, PD-L1 and PD-1 expression by TAMs also inhibits phagocytosis and tumor immunity revealing that immune checkpoint therapy functions through a direct effect on macrophage effector functions (Figure 2, left panel) [282,283]. Recently, the specific enrichment of M2-like CD163^+^CD33^+^PDL-1^+^ TAMs was associated with paradoxical boost in tumor growth in patients treated with immunotherapy, a phenomenon referred as “hyperprogression” [284]. Thus, TAMs may limit anti-PD-1 and other therapies by different means both in mice and humans. Altogether, these findings open substantial perspectives for improving immunotherapy efficacy including the combinations with strategies aimed at reeducating TAMs to limit their immunosuppressive functions or drug clearance capacity and help them eat cancer cells.

## 7. Conclusions

We have entered an exciting era of precision medicine with novel genetic and imaging modalities that should help to better stratify patient populations for which a battery of novel checkpoint blockade therapies will become available. Characterization of TME, especially its immune component, has provided an undoubtful value to our understanding of cancer development. Emerging evidences suggest that targeting macrophages and their metabolic reprogramming may have great therapeutic potential. Although, many large questions remain, there is no doubt that human clinical studies and the recent success of the CANTOS trial using an IL-1β inhibitory antibody will pave the way to the investigation of novel approaches to targeting not only traditional checkpoint blockade therapies but also immune checkpoint therapies to fight the residual burden of unmet need of NSCLC patients.
